# Association between Systemic Antioxidant Capacity and Retinal Vessel Diameters in Patients with Primary-Open Angle Glaucoma

**DOI:** 10.3390/life10120364

**Published:** 2020-12-20

**Authors:** Yuji Takayanagi, Yasuyuki Takai, Sachiko Kaidzu, Masaki Tanito

**Affiliations:** Department of Ophthalmology, Shimane University Faculty of Medicine, Izumo 693-8501, Japan; y.takayanagi1008@med.shimane-u.ac.jp (Y.T.); takai611@med.shimane-u.ac.jp (Y.T.); kecha@med.shimane-u.ac.jp (S.K.)

**Keywords:** redox status, oxidative stress, antioxidants, central retinal artery equivalent, central retinal vein equivalent, intraocular pressure, primary open-angle glaucoma, Diacron reactive oxygen metabolites (dROM), biological antioxidant potential (BAP), sulfhydryl (SH) test

## Abstract

The retinal vessel narrowing may be implicated in the pathogenesis of glaucoma; however, the association between systemic oxidative stress and retinal vessel diameter remains largely unknown. We examined the relationship between serum oxidative stress markers and retinal vessel diameters in eyes with primary open-angle glaucoma (POAG) and cataract, using central retinal artery equivalent (CRAE) and central retinal vein equivalent (CRVE). We included 66 eyes of 66 patients with POAG (37 men, 29 women; 65.4 ± 11.7 years) and 20 eyes of 20 patients with cataract (7 men, 13 women; 69.4 ± 9.0 years) as the controls. The CRAE (*p* < 0.0001), CRVE (*p* < 0.0001), and serum biological antioxidant potential (BAP) (*p* = 0.0419) were significantly lower in the POAG group compared to the controls. The BAP showed significant correlation both with CRAE (*ρ* = 0.2148, *p* = 0.0471) and systolic blood pressure (*ρ* = −0.2431, *p* = 0.0241), while neither Diacron reactive oxygen metabolites nor sulfhydryl test correlated with them. The multivariate analyses indicated that age, best corrected visual acuity, and BAP were independent factors for CRAE or CRVE. The present study suggested that lower systemic antioxidant capacity was significantly associated with the intraocular pressure-independent vascular narrowing in POAG patients. This study provided a novel insight into the pathophysiology of glaucoma and highlighted the clinical impact on systemic antioxidant treatment for patients with glaucoma.

## 1. Introduction

Primary open-angle glaucoma (POAG) is the most common type of glaucoma [1], which is characterized by chronic intraocular pressure (IOP) elevation and progressive loss of retinal ganglion cells [2]. The elevated IOP is a major responsible factor for the development of POAG [3]; however, the pathogenesis of POAG is multifactorial and not well recognized. Over recent decades, the association between retinal ganglion cell loss and narrowing of retinal arterial and venous vessel has been reported [4,5], and the assessment of retinal vessel diameter has gained popularity in patients with glaucoma.

Although arterial narrowing has been recognized as the most incipient sign of systemic hypertension for decades [6,7,8], other mechanisms have been discussed to elucidate the pathology of glaucoma. Several studies have focused on retinal vessel diameter [9,10], particularly in association with elevated IOP [11,12], retinal nerve fiber defect [13,14], and visual field impairment [15] in patients with glaucoma. However, few studies have examined the association between systemic oxidative stress and retinal vessel diameter in glaucomatous eyes.

Oxidative stress is thought to play an important role in the pathogenesis of glaucoma. Oxidative DNA damage can affect trabecular meshwork cells, which induces resistance to aqueous humor outflow and IOP elevation. Moreover, oxidative stress in retinal ganglion cells appears to be involved in the glaucomatous changes, and other mechanisms involving vascular alterations has not yet been clarified. We previously reported the relevance of oxidative stress in patients with glaucoma [16,17], and retinal vessel narrowing in eyes with pseudo-exfoliation syndrome [18]. Therefore, we hypothesized that oxidative stress may be implicated in the retinal vessel narrowing in glaucomatous eyes.

Here we examined the relationship between serum oxidative stress markers and retinal vessel diameters by estimation of central retinal artery equivalent (CRAE) and central retinal vein equivalent (CRVE) in eyes with POAG and cataract. The current study provided a novel insight into the IOP-independent pathogenesis of POAG in the context of oxidative stress and retinal vessel narrowing.

## 2. Materials and Methods

### 2.1. Subjects

The current study adhered to the tenets of the Declaration of Helsinki. The institutional review board of Shimane University Hospital approved the research (No. 20091119-1 and No. 20131216-1). All participants provided written informed consent. All subjects were recruited from the outpatient clinics of Shimane University Hospital. All patient information was anonymized. We included 66 eyes of 66 patients with POAG (37 men, 29 women; mean age ± standard deviation (SD), 65.4 ± 11.7) and 20 eyes of 20 patients with cataract (7 men, 13 women; mean age ± standard deviation (SD), 69.4 ± 9.0) as the controls. POAG was diagnosed based on bilateral open iridocorneal angles, characteristic glaucomatous optic changes such as enlargement of the optic disc cup or focal thinning of the neuro-retinal rim, corresponding with visual field defects identified by using the Humphrey Visual Field Analyzer Swedish Interactive Thresholding Algorithm central 30-2 program (Carl Zeiss Meditec, Jena, Germany) in at least one eye, and no evidence of secondary glaucoma. Control subjects fulfilled the conditions including no diagnosis of retinal diseases, no use of glaucoma medications, and the highest IOP of lower than 22 mmHg. In the control subjects, the eyes with excellent visibility of the optic disc margin and vessel borders were included in the analysis. In the POAG subjects, if both eyes were eligible, the eyes with better visibility of the optic disc margin and vessel border were included regardless of the progression of visual field loss. Eyes with fundus diseases other than POAG were excluded from the current study.

The patients underwent examinations including the measurement of systolic blood pressure, diastolic blood pressure, best corrected visual acuity (BCVA), and IOP obtained by Goldmann applanation tonometry (AT 900, Haag-Streit AG, Koeniz, Swizerland), slit-lamp microscopy (RO 5000, Rodenstock, Munich, Germany), gonioscopy using a two-mirror gonioscopy lens (Magna View Gonio, Ocular instruments, Bellevue, WA, USA), fundoscopy using a non-contact lens (Super Field, Volk Optical, Mentor, OH, USA), fundus photography (Nonmyd WX, Kowa Company, Aichi, Japan) and blood sampling. The measurements of systolic and diastolic blood pressure were conducted when the blood samples were taken.

### 2.2. Measurement of Systemic Oxidative Stress Markers

We collected venous blood samples in all participants. We used the collecting tube including thrombin and particles of silica, and the volume of each blood sample was 5 mL. The temperature during all procedures was maintained at 4 °C, including sample transportation and measurement of redox parameters in the laboratory setting. Serum samples were obtained by the centrifugation of the venous blood samples, and stored at 4 °C until the measurement. All blood analyses were performed using a free radical analyzer system (FREE Carpe Diem, Wismerll Company Ltd., Tokyo, Japan) that included a spectrophotometric device reader and thermostatically regulated mini-centrifuge; the measurement kits were optimized to the FREE Carpe Diem System, according to the manufacturer’s instruction [19]. Based on the manufacturer’s recommendation, all analyses were performed within 48 h of venous blood collection to avoid falsely high or low results. We conducted centrifugation for 10 min at 4977 rcf (3000 rpm) and 4 °C. The Diacron reactive oxygen metabolite (dROM) tests, biological antioxidant potential (BAP) tests, and sulfhydryl (SH) tests were performed, to analyze the serum levels of reactive oxygen metabolites, antioxidant capacity, and thiol antioxidant capacity, respectively.

The BAP test provides an estimate of the total antioxidant capacity of blood, which expressed as the ability to reduce ferric ions to ferrous ions. The decoloration occurred when the samples are instilled into the colored solution obtained by mixing a ferric chloride solution with a thiocyanate derivative solution, and the intensity of decoloration was measured by an absorbance change at 505 nm using a spectrophotometer [20,21]. The results are expressed in μmol/L of the reduced ferric ions.

The dROM test measures the amount of hydroperoxides that is related to the oxidation reaction of metabolites by free radicals. When the samples are dissolved in an acidic solution with a pH of 4.8, the hydroperoxides react and are converted to alkoxy and peroxy radicals in the presence of iron ions. These newly formed radicals oxidize an additive aromatic amine (N, N-diethyl-para-phenylen-diamine) and cause a formation of a relatively stable colored cation radical that is spectrophotometrically detectable at 505 nm [22,23]. The results are expressed in arbitrary units (U. Carr), one unit of which corresponds to 0.8 mg/L of hydrogen peroxide [22,23].

The SH test provides an estimate of the total thiol groups in the blood samples, using the modified Ellman method [24,25]. When the sample is added to a solution with 5,5-dithiobis-2-nitrobenzoic acid, thiols react and develop a stained complex that is spectrophotometrically detectable at 405 nm and is proportional to their concentration according to the Beer–Lambert law [21,23]. The results are expressed as μmol/L of the SH groups.

### 2.3. Measurement of Retinal Vessel Calibers

All fundus photographs were taken by well-trained examiners. The retinal vessel diameters were measured on digitized 40-degree fundus photographs centered on the optic disc using the IVAN software (Department of Ophthalmology and Visual Science, University of Wisconsin, Madison, WI, USA) [26]. A non-blinded well-trained researcher measured all individual retinal vessels that were 25 μm or larger and passed completely through a circumferential zone of a 0.5 to 1 disc diameter from the optic disc margin. The six widest arteriolar and venular diameter then were defined as the CRAE or the CRVE using the previously reported formula [27,28]. The magnification of the optic media was corrected with the Littmann formula [29].

### 2.4. Statistical Analysis

We used the Mann–Whitney U test for comparisons of continuous data between the two groups, including age, systolic blood pressure, diastolic blood pressure, BCVA, IOP, CRAE, CRVE, serum BAP, dROM, and SH levels, and Fisher’s exact probability test for those of categorical data, i.e., sex. Spearman’s correlation test was performed on clinical parameters, i.e., age, systolic blood pressure, diastolic blood pressure, BCVA, IOP, CRAE, CRVE, BAP, dROM, and SH. To determine the independent factors associated with the retinal vessel diameter, we also performed multivariate logistic regression analyses with CRAE or CRVE as the response variables and with the covariates of age, sex, systolic blood pressure, BCVA, IOP, and one of the redox parameters including BAP, dROM, and SH. All statistical analyses were calculated using the JMP Pro statistical software version 14.2 (SAS Institute, Inc., Cary, NC, USA). All statistical analyses were performed by two-sided. *P* value of 0.05 was considered statistically significant. The data are expressed as the means ± SD for continuous variables and in numbers and percentage for categorical variables. For the statistical analyses, the decimal BCVA recorded was converted into the logarithm of the minimum angle of resolution (LogMAR). Counting fingers, hand motions, light perception, and no light perception were regarded as decimal visual acuity of 0.0025, 0.002, 0.0016, and 0.0013, respectively [30].

## 3. Results

The demographic subject data, including age, sex, systolic blood pressure, diastolic blood pressure, BCVA, IOP, and the number of glaucoma medications are shown in Table 1. The BCVA (LogMAR) was significantly worse in patients with POAG (0.03 ± 0.34, *p* < 0.0001) than in the control group (0.28 ± 0.44), and the other parameters did not differ significantly.

Table 2 demonstrates the comparison of retinal vessel diameters and serum oxidative stress markers between the controls and the POAG groups. The CRAE (93.9 ± 1.8 μm, *p* < 0.0001) and CRVE (140.9 ± 21.4 μm, *p* < 0.0001) in patients with POAG were significantly smaller than in the controls (134.3 ± 3.3 μm and 194.0 ± 25.9 μm, respectively). The serum BAP levels (1892.9 ± 236.4 μmol/L, *p* = 0.0419) in the POAG group were significantly lower compared to the controls (2137.6 ± 446.8 μmol/L). The dROM and serum SH values were not significantly different between the two groups.

Table 3 summarizes the correlations among clinical parameters including age, systolic blood pressure, diastolic blood pressure, BCVA, IOP, CRAE, CRVE, BAP, dROM, and SH. In the systemic oxidative stress markers, the BAP showed a significant correlation both with CRAE (*ρ* = 0.2148, *p* = 0.0471) and systolic blood pressure (*ρ* = −0.2431, *p* = 0.0241), while neither dROM nor SH correlated with them. The CRAE significantly correlated with age (*ρ* = −0.2637, *p* = 0.0141) and BCVA (*ρ* = 0.2900, *p* = 0.0068). Similarly, the CRVE showed a significant correlation with age (*ρ* = −0.2559, *p* = 0.0174) and BCVA (*ρ* = 0.3499, *p* = 0.0010). Additionally, there was a significant correlation between SH and age (*ρ* = −0.2515, *p* = 0.0195).

Table 4 demonstrates multiple linear regression models of factors potentially associated with CRAE and CRVE, which included age, sex, systolic blood pressure, BCVA, IOP, and either one of systemic oxidative stress markers (i.e., BAP, dROM, and SH). The models including BAP indicated that the age (*p* = 0.0001), BCVA (*p* = 0.0049), and BAP (*p* = 0.0019) were independent variables that were significantly associated with CRAE. Similarly, the age (*p* = 0.0097), BCVA (*p* = 0.0018), and BAP (*p* = 0.0157) were significantly associated with CRVE. In the other models, the dROM and SH were not the independent factor for determining CRAE or CRVE.

## 4. Discussion

This study was designed to investigate the association between serum oxidative stress parameters and retinal vessel diameters, and to elucidate the potential role of systemic oxidative stress in patients with POAG. Overall, the current study suggested two important clinical findings. First, the CRAE and CRVE were significantly lower in patients with POAG, who did not have significantly higher IOP compared to controls. Second, significantly lower BAP was found in the POAG group, and lower BAP was independently associated with lower CRAE and CRVE.

First, our results revealed significantly lower CRAE and CRVE in patients with POAG than in the controls. Several previous studies reported the peripapillary retinal vascular narrowing in patients with glaucoma [31,32,33], which were generally in line with our results. According to these previous reports, the narrowing of retinal blood vessels in glaucoma might be a part of disease process due to elevated IOP and structural changes of the optic cup, rather than a cause of optic nerve fiber loss. However, other cohort study revealed that patients with narrower retinal blood vessels had approximately fourfold risk for developing open-angle glaucoma [34]. Therefore, we speculated that the retinal vessel narrowing may be implicated in the pathogenesis of open-angle glaucoma.

It is important to note that the IOP were not statistically significant between the POAG group and the control group; nevertheless, the significantly different CRAE and CRVE between two groups were found in this study. Previous evidence elucidated that narrower retinal arteriolar and venular changes, which is independent of elevated IOP, were associated with structural changes of the optic cup in glaucomatous eyes [35]. This was clearly consistent with our findings, and this evidence suggested that IOP-independent mechanisms may be implicated with the chronic retinal vascular narrowing in glaucomatous eyes.

The second clinical suggestion we provided here is that significantly lower BAP was observed in patients with POAG, and lower BAP was independently related to narrower CRAE and CRVE in the multivariate analyses. Systemic oxidative stress including lower BAP has been thought to play an important role in the pathology of open-angle glaucoma [36,37,38]. We previously reported the significant correlation between BAP and IOP in patients with POAG, suggesting that lower systemic antioxidant capacity may be involved in the pathogenesis of glaucoma due to its impact on IOP elevation [39]. Other previous study demonstrated that lower BAP was associated with decreased ocular blood flow in patients with normal tension glaucoma [40]. These pieces of evidence are consistent with the results of this study, and the current study further extended the concept that lower systemic antioxidant capacity might contribute constitutively to the pathophysiological changes in glaucomatous patients, via IOP-independent mechanisms including the retinal vascular narrowing.

One possible explanation for this is that oxidative stress may play an important role for the retinal vascular narrowing via a dysfunction of retinal vascular tone in glaucoma. The narrowing of the arteriolar caliber was caused by functional stenosis of the vascular lumen, rather than thickening of the vascular wall due to atherosclerosis [41], and a decrease in nitric oxide marker and increase in endothelin level were found in glaucomatous eyes [42]. This evidence suggested that narrowing of the retinal vessels may result from dysregulation of vascular tone via endogenous vasodilators such as nitric oxides. Very few studies have examined the relationship between oxidative stress and retinal vascular diameters. One previous study demonstrated that the glutathione peroxidase activity, which is a biomarker of oxidative stress regulations, was associated with the retinal arteriolar caliber in elderly individuals without a history of coronary artery disease or stroke [43]. Glutathione peroxidase deficiency has been reported to be related with an dramatic increase in extracellular peroxide-related oxidants and decrease in bioavailable nitric oxide [44]. Therefore, it is biologically plausible that systemic oxidative stress can induce the vascular tone dysfunction leading to the narrowing of retinal vessels.

It is also important to emphasized that the serum SH levels were significantly associated with age, and the age is independent factor for CRAE and CRVE in this study. Our previous report also showed the correlation between SH values and age. Additionally, an age-related decline in glutathione levels has been observed in several studies [45,46]. Previous reports indicated that aging was related to the vessel narrowing [47,48]; however, we hypothesized that retinal vessels may undergo more advanced pathological changes in patients with glaucoma, when exposed to the aggravating factors such as oxidative stress.

Lastly, there were several limitations to the present study that are noteworthy, as they may affect generalization of our findings. First, our study has the same limitations of any retrospective study in being neither controlled nor randomized. Especially, the IOP of POAG groups was the value of under use of glaucoma medications; thus, our study does not exclude the possible roles of the systemic oxidative stress on IOP elevation. Second, the senile aged population and the comparison to the cataract controls with worse BCVA could limit the generalization of our results. Although the better BCVA was independent factor for narrower CRAE or CRVE in the current study, low visual acuity due to cataract might affect our results. Third, small sample size of this study had insufficient power to detect significant association among all parameters. Fourth, we did not confirm the uptake of antioxidant nor vitamin supplements and measured the antioxidant parameters with a single method. This can potentially affect the data reliability. Finally, we have analyzed our data in comparison between prostaglandin- and non- prostaglandin users, β-blocker- and non-β-blocker users, and CAI and non-CAI users; however, no significant difference was observed between each comparison in terms of CRAE, CRVE, and oxidative stress markers including BAP (data not shown). Most participants were prescribed the multiple glaucoma medications; therefore, it is difficult to examine the impact of glaucoma medications and this warrants further study. Despite these limitations, our study has many strengths, including clinically generalizable sample of patients, the precise measurement of retinal vascular diameters, and comprehensive assessment of systemic redox parameters.

## 5. Conclusions

In conclusion, the present study suggested that lower systemic antioxidant capacity was significantly associated with the narrowing of retinal vascular calibers in POAG patients. This study provided a novel insight into the pathophysiology of glaucomatous optic neuropathy via oxidative stress-induced vessel narrowing and highlighted the possible clinical impact on systemic antioxidant treatment for patients with glaucoma. The present findings warrant further research to investigate the precise biological mechanisms for clarifying the relationship between oxidative stress and retinal vessel narrowing in glaucoma.

## Figures and Tables

**Table 1:** Demographic subject data.

Parameters	Control	POAG	*p*-Value ^a^
N	20	66	
Age (years)			
Mean ± SD	69.4 ± 9.0	65.4 ± 11.7	0.3388
range	55.0–88.0	24.0–84.0	
Sex			
Men, n (%)	7 (35.0)	37 (56.0)	0.1278
Women, n (%)	13 (65.0)	29 (44.0)	
sBP (mmHg)			
Mean ± SD	135.9 ± 17.9	139.3 ± 21.9	
range	105.0–172.0	102.0–191.0	
dBP (mmHg)			
Mean ± SD	78.3 ± 11.1	80.8 ± 12.8	0.4738
range	62.0–95.0	52.0–110.0	
BCVA (LogMAR)			
Mean ± SD	0.28 ± 0.44	0.03 ± 0.34	<0.0001 **
range	−0.08–1.00	−0.08–1.70	
IOP (mmHg)			
Mean ± SD	14.7 ± 2.8	15.9 ± 6.7	0.8492
range	11.0–22.0	6.0–52.0	
Glaucoma medication (n)			
Mean ± SD		1.5 ± 1.2	
range		0.0–5.0	
Types of glaucoma medication			
Prostaglandins, n (%)		51 (77.2)	
β-blockers, n (%)		30 (45.5)	
CAI, n (%)		15 (22.7)	
Cholinergics, n (%)		1 (1.5)	

^a^ Comparison between the controls and the POAG groups by using the Mann–Whitney U test for continuous data and by using Fisher’s exact probability test for categorical data. The ** corresponds to the significance level at 1% (*p* < 0.01). N, number of participants; SD, standard deviation; POAG, primary open-angle glaucoma; sBP, systolic blood pressure; dBP, diastolic blood pressure; BCVA, best-corrected visual acuity; logMAR, logarithm of the minimum angle of resolution; IOP, intraocular pressure; CAI, carbonic anhydrase inhibitors.

**Table 2:** Comparison of retinal vessel diameters and serum oxidative stress markers.

Parameters	Control	POAG	*p*-Value ^a^
N	22	66	
CRAE (μm)			
Mean ± SD	134.3 ± 3.3	93.9 ± 1.8	<0.0001 **
range	93.0–159.5	65.1–127.6	
CRVE (μm)			
Mean ± SD	194.0 ± 25.9	140.9 ± 21.4	<0.0001 **
range	122.7–241.4	96.7–186.8	
serum BAP (μmol/L)			
Mean ± SD	2137.6 ± 446.8	1892.9 ± 236.4	0.0419 *
range	1618.7–3031.0	1342.8–2528.0	
serum dROM (U. Carr)			
Mean ± SD	333.6 ± 55.8	348.4 ± 60.0	0.5031
range	206.0–439.0	255.0–505.0	
serum SH (U.Carr)			
Mean ± SD	651.5 ± 80.3	639.0 ± 95.3	0.4252
range	504.0–787.0	411.0–884.0	

^a^ Comparison between the controls and the POAG groups by using the Mann–Whitney U test for continuous data. The * and ** correspond to the significance levels at 5% (*p* < 0.05) and 1% (*p* < 0.01), respectively. N, number of participants; SD, standard deviation; POAG, primary open-angle glaucoma; CRAE, central retinal artery equivalent; CRVE, central retinal vein equivalent; BAP, biological antioxidant potential; dROM, Diacron reactive oxygen metabolites; SH, sulfhydryl.

**Table 3:** Correlations among clinical parameters.

*ρ*/*p*-Value	Age	sBP	dBP	BCVA	IOP	CRAE	CRVE	BAP	dROM	SH
Age	−	0.1190	−0.0727	0.0896	−0.0129	−0.2637	−0.2559	0.0641	−0.0018	−0.2515
sBP	0.2752	−	0.6705	−0.1148	0.1225	−0.1361	−0.1477	−0.2431	0.0731	−0.1858
dBP	0.5060	<0.001 **	−	−0.1894	0.0008	−0.1229	−0.1387	−0.0147	0.1275	−0.0798
BCVA	0.4122	0.2928	0.0807	−	0.0143	0.2900	0.3499	0.1756	0.2039	0.1349
IOP	0.9065	0.2611	0.9939	0.8960	−	−0.0686	−0.0480	−0.0267	0.0197	−0.1223
CRAE	0.0141 *	0.2116	0.2594	0.0068 *	0.5303	−	0.9040	0.2148	−0.0991	0.1500
CRVE	0.0174 *	0.1747	0.2027	0.0010 *	0.6608	<0.0001 *	−	0.1658	−0.1031	0.2077
BAP	0.5575	0.0241 *	0.8928	0.1059	0.8073	0.0471 *	0.1270	−	0.1446	0.1314
dROM	0.9869	0.5036	0.2422	0.0597	0.8571	0.3642	0.3448	0.1841	−	−0.1965
SH	0.0195 *	0.0868	0.4651	0.2157	0.2620	0.1681	0.0550	0.2278	0.0697	−

Correlation coefficient (*ρ*) and *p*-values for each pair of groups, calculated by using Spearman’s correlation test. The * and ** correspond to the significance levels at 5% (*p* < 0.05) and 1% (*p* < 0.01), respectively. sBP, systolic blood pressure; dBP, diastolic blood pressure; BCVA, best-corrected visual acuity; IOP, intraocular pressure; CRAE, central retinal artery equivalent; CRVE, central retinal vein equivalent; BAP, biological antioxidant potential in serum; dROM, Diacron reactive oxygen metabolites; SH, sulfhydryl.

**Table 4:** Multiple linear regression analyses for CRAE and CRVE.

	CRAE	95% CI	*p*-Value	CRVE	95% CI	*p*-Value
	Estimate			Estimate		
Entire model			0.0003 **			<0.0001 **
Age (/year)	−0.5555	51.14, 149.90	0.0001 **	−0.7702	−1.34, −0.19	0.0097 **
Women (/men)	0.3326	−0.96, −0.15	0.8832	−1.6096	−8.04, 4.82	0.6198
sBP (/mmHg)	0.0100	−4.16, −4.82	0.9271	−0.0177	−0.33, 0.29	0.9100
BCVA (/LogMAR)	20.1896	6.32, 34.06	0.0049 **	32.1612	12.29, 52.02	0.0018 **
IOP (/mmHg)	−0.6185	−1.35, 0.11	0.0963	−0.8436	−1.89, 0.20	0.1129
BAP (μmol/L)	0.0237	0.01, 0.04	0.0019 **	0.0261	0.01, 0.05	0.0157 *
Entire model			0.0071 **			0.0063 **
Age (/year)	−0.4277	−0.84, −0.01	0.0436 *	−0.6285	−1.21, −0.05	0.0348 *
Women (/men)	2.1107	−2.53, 6.75	0.3682	0.4074	−6.11, 6.92	0.9012
sBP (/mmHg)	−0.0595	−0.28, 0.16	0.5933	−0.0920	−0.40, 0.22	0.5563
BCVA (/LogMAR)	21.2827	6.67, 35.88	0.0048 **	33.5454	13.05, 54.03	0.0017 **
IOP (/mmHg)	−0.5729	−1.34, 0.19	0.1405	−0.7898	−1.86, 0.28	0.1417
dROM (U.Carr)	−0.0627	−0.14, 0.02	0.1144	−0.0777	−0.19, 0.03	0.1629
Entire model			0.0194 *			0.0140 *
Age (/year)	−0.411	−0.87, −0.01	0.0451 *	−0.6395	−1.24, −0.03	0.0380 *
Women (/men)	1.7285	−2.96, 6.42	0.4662	−0.0851	−6.65, 6.48	0.9795
sBP (/mmHg)	−0.0793	−0.30, 0.14	0.4842	−0.1150	−0.43, 0.20	0.4682
BCVA (/LogMAR)	20.0942	5.27, 34.91	0.0085 **	31.9790	11.21, 52.70	0.0029 **
IOP (/mmHg)	−0.6076	−1.39, 0.17	0.1276	−0.8260	−1.92, 0.27	0.1385
SH (U.Carr)	−0.0044	−0.05, 0.05	0.8670	−0.0023	−0.07, 0.07	0.9498

Multiple linear regression analyses of factors potentially associated with CRAE and CRVE, which include age, sex, sBP, BCVA, IOP, and BAP. P values are calculated with the use of likelihood ratio test. The * and ** correspond to the significance levels at 5% (*p* < 0.05) and 1% (*p* < 0.01), respectively. SD, standard deviation; CI, confidence interval; sBP, systolic blood pressure; BCVA, best-corrected visual acuity; logMAR, logarithm of the minimum angle of resolution; IOP, intraocular pressure; BAP, biological antioxidant potential; dROM, Diacron reactive oxygen metabolites; SH, sulfhydryl.
